# Breaking bad news in Medecine : Tunisian Trainees’ feelings

**DOI:** 10.1192/j.eurpsy.2023.2383

**Published:** 2023-07-19

**Authors:** L. Sahli, Y. Zgueb, A. aissa, U. Ouali

**Affiliations:** 1 Razi Hospital, Manouba, Tunisia

## Abstract

**Introduction:**

The announcement of bad news to patients is a challenging task for physicians.

**Objectives:**

The aim of our study is to evaluate the impact of a simulation experience of telling bad news to patients on Tunisian medical trainees.

**Methods:**

A prospective and multicenter study was conducted in two different hospitals in Tunisia. The duration of the study was two weeks. Pre-prepared questionnaires evaluating the impact of a simulation experience of the announcement of a critical illness diagnosis were handed to trainees enrolled in the faculty of Medecine in Tunis.

**Results:**

Forty trainees were included in the study. Average age was 28,1 years old with a feminine predominance (75%). Thirteen trainees role-played the clinician announcing the bad news. The median duration of the simulated interview was eight minutes. During the moment of the diagnosis announcement, twelve trainees reported feeling stressed, 6 of them felt uncomfortable and 7 felt empathic. Five trainees were in difficulty, two felt at ease and two felt neutral. During the whole interview, all the trainees reported they felt stressed and uncomfortable. Regarding their feelings at the end of the interview, only two reported they felt relieved at the end of the interview. Three trainees felt angry with themselves. Two felt angry with the patient or his family members. Eight felt upset and four were in tears. Four reported having no feelings.

**Conclusions:**

Telling bad news is a difficult moment in daily practice. Special trainings need to be implemented in the medical education program in order to prepare future doctors to this task.

**Disclosure of Interest:**

None Declared

